# Liver X Receptors and Male (In)fertility

**DOI:** 10.3390/ijms20215379

**Published:** 2019-10-29

**Authors:** Sheba Jarvis, Catherine Williamson, Charlotte L Bevan

**Affiliations:** 1 Department of Surgery and Cancer, Imperial College London, London W12 0NN, UK; charlotte.bevan@imperial.ac.uk; 2 Department of Women and Children’s Health, King’s College London, London SE1 9UL, UK; catherine.williamson@kcl.ac.uk

**Keywords:** liver X receptors, testis, infertility, steroidogenesis, oxysterols

## Abstract

Liver X receptors (LXRs) are ligand-dependent transcription factors acting as ‘cholesterol sensors’ to regulate lipid homeostasis in cells. The two isoforms, LXRα (NR1H3) and LXRβ (NR1H2), are differentially expressed, with the former expressed predominantly in metabolically active tissues and the latter more ubiquitously. Both are activated by oxidised cholesterol metabolites, endogenously produced oxysterols. LXRs have important roles in lipid metabolism and inflammation, plus a number of newly emerging roles. They are implicated in regulating lipid balance in normal male reproductive function and may provide a link between male infertility and lipid disorders and/or obesity. Studies from Lxr knockout mouse models provide compelling evidence to support this. More recently published data suggest distinct and overlapping roles of the LXR isoforms in the testis and recent evidence of a role for LXRs in human male fertility. This review summarises the current literature and explores the likely link between LXR, lipid metabolism and male fertility as part of a special issue on Liver X receptors in International Journal of Molecular Sciences.

## 1. Introduction

Liver X receptors (LXRs) are transcription factors that act as master regulators of lipid homeostasis by functioning as ‘cholesterol sensors’ [1]. They are part of the nuclear receptor (NR) superfamily with two isoforms, LXRα (NR1H3) and LXRβ (NR1H2), encoded by distinct genes located on chromosome 11 and 19, respectively [2,3].

LXRs are ligand activated and upon activation form permissive heterodimers with Retinoid X receptors (RXRs) [4]. They also have an important relationship with another nuclear receptor, Farnesoid X receptor (FXR), and together they play important roles in cholesterol-bile acid homeostasis [4].

There is an increasing interest in the roles of LXRs outside the enterohepatic system, where they were initially characterised, with evidence for regulatory effects in macrophages, brain, adipose cardiorespiratory and endocrine systems. These emerging roles are in cancer, autoimmune and neurodegenerative diseases as well as inflammation [5,6]. It is also increasingly apparent that LXRs are critical to male reproduction and this article focuses on their roles in the testis and epididymis, sites of spermatogenesis and post-testicular maturation, respectively.

## 2. LXR Structure, Function and Expression

LXRs are referred to as a Class II nuclear receptors since they are located in the nucleus where, regardless of ligand binding, they bind LXR response elements (LXREs) in the promotor region of their target genes and recruit corepressors, causing repression of target gene expression. LXR co-repressors include silencing mediator of retinoic acid and thyroid receptor (SMRT) and nuclear receptor corepressor (NCoR) [7,8]. Upon ligand binding, conformational change in the protein leads to a shift to coactivator recruitment and target gene expression.

LXRα and LXRβ share ~77% amino acid sequence homology in the ligand binding domain [9] and each have 4 functional domains. These are (i) an N’ terminal domain, containing coactivator binding sites and a transcriptional function (AF-1) (ii) the DNA-binding domain, a highly conserved area with the binding activity mediated by two zinc fingers, (iii) a hinge region allowing receptor flexibility and corepressor recruitment and (iv) a C’ terminal region with the AF-2 domain which regulates transcription via interactions with co-activators and co-repressors after ligand binding. The ligand binding domain contains α helices organised around a central ligand binding hydrophobic pocket [10,11]. The LXREs, bound by LXR/RXR heterodimers, are referred to as DR-4 binding sites and are usually comprised of a repeated sequence of 5-AGGTCA-3 separated by a 4 nucleotide spacer [2,12]. Although this is a common LXRE, recently other LXR binding sites have been identified from ChIP-seq studies [12,13,14].

Interestingly, other modulatory roles for LXRs are increasingly described as involving post-translational modification processes including SUMOylation, acetylation, phosphorylation, ubiquitination and O-GlcNacylation, but there is a paucity of data in these areas and in particular with regards to the potential for non-genomic effects of LXRs [5,15].

In terms of pattern of expression, LXRβ is ubiquitously expressed throughout the body, whereas LXRα (NR1H3) is most highly expressed in metabolically active tissues such as the liver, kidney, adrenal glands, macrophages and the intestine [16]. In the testis, there is widespread expression of LXRs with differential expression in the various germ and somatic cell types [17,18] whilst the cells of the epididymis express both LXR isoforms.

## 3. LXRs Are Activated by Oxysterols and Are Central Regulators for Lipid Metabolism

Oxysterols are oxidised cholesterol metabolites which act as natural ligands for LXR receptors; they have a side chain hydroxyl group which is essential for LXR activation [19]. Oxysterols that bind to and activate LXR, with varying affinity and potency, respectively, include: 20(S)-, 22(R)-, 24(S)-, 25-, (25R),26-hydroxycholesterol; 24(S),25-epoxycholesterol, cholestenoic acid as well as the cholesterol intermediates: desmosterol, follicular fluid meiosis-activating sterol (FF-MAS), testis meiosis-activating sterol (T-MAS) [3,20,21,22]. Many of the LXR activating oxysterols are present in the reproductive tract [23,24,25]; however, there is still a limited amount of information on the role of oxysterols, with tissue specific and potentially cell specific variation in oxysterol production, making it problematic to predict which are the most physiologically relevant. This, plus the varying potency of endogenous ligands and their potential to activate other nuclear receptors [11,26], is likely the reason that often under experimental conditions, synthetic LXR ligands - notably T0901317 and GW3965 - are used. Again, however, although these are relatively specific for LXR, data should be interpreted with the caveat that they may vary in affinities for LXR isoforms and potentially different effects to endogenous oxysterols.

In the female, FF-MAS (produced from lanosterol and catalysed by lanosterol 14α-demethylase protein complex) has been shown to be important for oocyte meiosis and survival and is associated with successful implantation [27,28,29]. FF-MAS has been shown to stimulate a putative receptor within oocytes, for which LXRα may be a candidate [30,31]. FF-MAS is synthesized in large amounts by granulosa cells of the maturing ovarian follicle but whether similar findings are seen in the male counterpart, the Sertoli cell of the testis, requires more investigation. However, T-MAS (converted from FF-MAS by sterol 14α-reductase) has been identified in the bull and mouse testis and is potentially important for meiosis [32,33,34]. Furthermore, lanosterol 14α-demethylase, important in oxysterol production, shows stage-specific expression in developing spermatids [34]. Also, 25-hydroxycholesterol is able to activate the LXR receptors and is produced by rat testicular macrophages and used by Leydig cells in androgen production [35,36]. More recently this oxysterol and (25R),26-hydroxycholesterol (previously denominated 27-hydroxycholesterol) were identified in human sperm, with 25-hydroxycholesterol observed to mediate the acrosome reaction necessary for normal sperm physiology and fertilization [22,25].

Cholesterol homeostasis is strictly regulated within cells, and the main source of cholesterol is dietary with cholesterol uptake into cells via scavenger receptor, class B type (SR-B1) with SR-B1 overexpression states associated with high cellular esterified cholesterol levels [37] or due to de novo cholesterol biosynthesis [38]. The cholesterol biosynthesis pathways require acetyl CoA, and a series of enzymatic reactions occur during sterol metabolism which, via mevalonate, culminate in cholesterol production.

**Figure 1:**
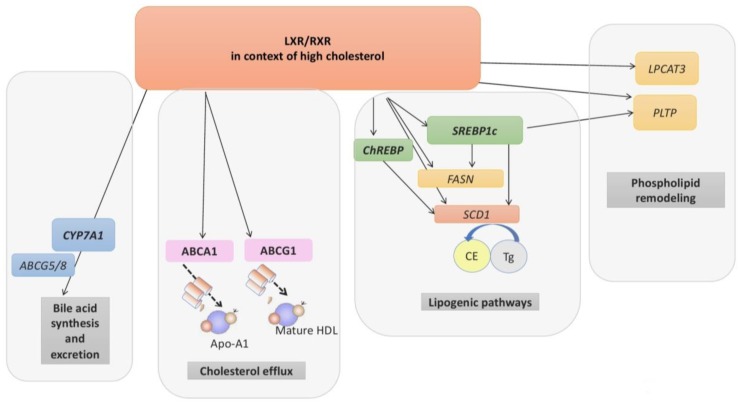
The roles of LXRs in lipid metabolism. LXRs regulate hepatic cholesterol elimination by upregulating CYP7A1 as well as excreting cholesterol via ATP binding cassette transporters ABCG5/8 (typically in hepatobiliary system). They also facilitate reverse cholesterol transport by regulating cholesterol efflux from peripheral tissues and cells (e.g., typically macrophages, Sertoli cells of the testis) where ABCA1 and ABCG1 transport cholesterol to APO-A1-HDL and mature HDL respectively. LXRs regulate lipogenesis (usually via hepatic LXRα) with upregulation of SREBP1c, FASN and SCD-1. ChREBP is also able to activate SCD-1 but has a role in carbohydrate metabolism. Finally, LXRs regulate phospholipid remodelling through direct activation of LPCAT3, a crucial enzyme in this process which facilitates the turnover of PUFAs (shown to occur in macrophages, liver, intestine), which will affect membrane phospholipid and allows cells to become resistant to sterol mediated cellular stress. LXRs and the SREBP1 axis are also important for the activation of PLTP which facilitates the production of nascent VLDL. CE, cholesteryl esters; Tgs, triglycerides.

Maintenance of cellular and systemic sterol levels is an essential homeostatic process, and LXRs act as critical sterol sensors but also regulate fatty acid and phospholipid metabolism [39]. One method of cholesterol elimination in the liver is by induction of *CYP7A1;* this encodes the enzyme cholesterol 7α-hydroxylase which catabolises cholesterol into bile acids for excretion via the LXR-upregulated ATP binding cassette (ABC) transporters: ABCG5 and ABCG8 [40,41]. Another means of tightly regulating cellular cholesterol levels is reverse cholesterol transport (RCT), whereby excess cholesterol is effluxed from peripheral tissues and returned to the liver via high-density lipoproteins (HDL). LXRs regulate cellular cholesterol efflux transporters important for RCT, ABCA1 and ABCG1 [42,43]. ABCA1 controls transfer of cholesterol and phospholipids from plasma membranes to pre-HDL or to lipid-poor APOA-1 molecules [44] and is assisted by ABCG1 [45] (Figure 1).

LXRs also control de novo lipogenesis via induction of sterol regulatory element binding protein 1c (SREBP1c), a major gatekeeper of lipogenesis. The SREBP family has 3 members, which are part of a family of basic helix-loop-helix leucine zipper transcription factors that regulate key lipogenic genes, including fatty acid synthase (*FASN*) and stearoyl coenzyme A desaturase 1 (*SCD1*); these are also directly targeted by LXRα and LXRβ. Carbohydrate metabolism is also implicated; LXR targets carbohydrate response element binding protein (ChREBP) and is another example of how LXRs influence lipogenesis [39,46] (Figure 1).

LXRs are also involved in phospholipid remodelling processes and LXRα induces expression of the gene encoding the enzyme lysophosphatidylcholine acyltransferase 3 (LPCAT3), involved in phospholipid remodelling in response to changing sterol levels [47,48]. LPCAT3 catalyses incorporation of polyunsaturated fatty acids at the *sn-2* site of lysophospholipids, which affects the fluidity and the curvature of the membranes and protects against high sterol related stress at cell membranes [39]. Phospholipid transfer protein (PLTP) is also a direct LXR target, which transfers phospholipids between lipoprotein particles [39,49,50]. Together, LXR and SREBP1c activate PLTP which produces nascent VLDL (very low density lipoprotein) particles [51] (Figure 1).

The roles of LXRs in cholesterol homeostasis were revealed largely by use of the transgenic LXR knockout mice. *Lxrα* -/- mice fed a high fat diet develop hepatic steatosis, from deposition of cholesteryl esters due to failure to upregulate hepatic *Cyp7a1,* which would normally lead to conversion of cholesterol into bile acids [40,52]. More recently, a liver specific knockout revealed that hepatic LXRα modulates lipoprotein particle number and intestinal LXR activity is likely responsible for increasing HDL cholesterol [53].

## 4. Cholesterol, Somatic Cells and Germ Cell Maturation

Spermatogenesis is a complex but highly ordered process of male germ cell maturation taking place within the seminiferous tubules [54]. Once produced, spermatozoa exit the seminiferous tubules and transit through the epididymis, an accessory organ crucial for post testicular maturation. To function normally, the testis requires an orchestration of events which include germ cell proliferation, differentiation, apoptosis and critically communication between germ cell and somatic cell. This takes place between germ cells and Sertoli cells, as well as complex paracrine signalling between the somatic cells of the testis [55].

In the testis, cholesterol serves as a vital fuel for androgen production (during steroidogenesis), as well as in the maintenance of cell membranes and, along with fatty acids, is a potential energy source for Sertoli cells [56,57,58]. For germ cells, cholesterol is an important for membrane remodeling during spermatogenesis, and spermatocytes are capable of undergoing de novo lipogenesis and cholesterol uptake with a surge of cholesterol utilisation during meiosis [59]; it is not clear if this also occurs in other germ cell subtypes.

Cholesterol is the most abundant sterol in germ cell membranes, which undergo extensive remodelling during germ cell maturation in the testis and also in the epididymis. The ratios of different lipids within the spermatozoal membrane are important for normal functioning and subsequent signalling events and they are rich in polyunsaturated fatty acids (PUFAs), necessary for motility, capacitation and sperm-egg fusion [60,61].

Sertoli cells are the ‘nurse’ cells for developing germ cells and their numbers are critical for spermatogenesis [62]. They are responsible for maintenance of the blood testis barrier (BTB), producing extracellular matrix, transport proteins, cytokines, androgen binding proteins (ABP) with many other functions including lipid storage and efflux [38]. Sertoli cells supply cholesterol and fatty acids needed for germ cell maturation [56,57] and although they can undergo de novo lipogenesis this is not sufficient for the amount of lipid required for spermatogenesis [38]. Thus, uptake of circulating cholesterol into Sertoli cells via SR-B1 allows HDL-derived cholesteryl esters to be used as a major lipid source [63] and they may also use lipid droplets in this respect.

Despite the need for large amounts of lipid, Sertoli cells must also have mechanisms whereby intracellular lipid levels can be finely tuned and they express high levels of jjABCA1 and ABCG1, key players in lipid efflux [64]. Any imbalance in lipid homeostasis may culminate in excessive lipid droplet (LD) deposition within the Sertoli cell cytoplasm, which can affect function. Excessive large LDs may cause mechanical dysfunction through altering cytoskeleton, and BTB disruption, affecting germ cell survival and maturation during spermatogenesis [65,66].

Leydig cells also utilise large amounts of cholesterol for androgen production; regulation of steroidogenesis in these cells is highly complex and requires multiple enzymatic reactions. Although the Leydig cells can synthesise cholesterol de novo in the endoplasmic reticulum, this is not sufficient for steroidogenesis and other sources are required; these include utilisation of lipid droplets (LDs) of cholesterol esters and uptake of circulating lipoproteins, e.g., HDL via SR-B1and also uptake of low density lipoprotein [67]. LXRs (as well as other nuclear receptors including FXR, small heterodimer partner (SHP) and steroidogenic factor 1 (SF-1) have been implicated in the regulation of steroidogenesis [38,68].

When considering the post testicular maturation of sperm, marked changes in lipid composition are crucial for fertility. The passage of sperm through the epididymis is a crucial form of quality control of eradicating abnormal sperm but also facilitating modifications to the sperm membrane by altering the cholesterol content [34]. Cholesterol has a major effect on sperm membrane fluidity and cholesterol efflux is required for sperm to acquire appropriate characteristics necessary for capacitation. Therefore, any abnormality in cholesterol metabolism or phospholipid regulation could potentially impact male fertility [38,69]. Higher dietary cholesterol intake modifies spermatozoa quality in a negative manner in rodent studies [70] although more recently in human studies of seminal plasma, analysis suggests higher serum cholesterol levels are associated with favourable semen parameters [71,72]. Ultimately, a delicate balance, which is tightly regulated in the testis, is likely to be important.

## 5. LXRs Are Important for Maintenance of Male Fertility

LXRα and LXRβ are expressed in the mouse and human testes [17,73,74] and an understanding of testicular LXRs has originated from studies using knockout mice where either LXR isoform (*Lxrα* -/- or *Lxrβ* -/-) or both LXR isoforms (*Lxrαβ* -/-) are ablated [38,68]. In the mouse, Leydig cells express mainly LXRα, and Sertoli cells express LXRβ, whilst both isoforms are expressed in germ cells [18]. Early work suggested redundancy of LXR isoforms since *Lxrα* -/- or *Lxrβ* -/- mice are fertile whilst in contrast, *Lxrαβ* -*/*- mice have a severe infertility phenotype [18,66,73]. *Lxrαβ* -*/*- male mice are sub-fertile by 4–5 months of age, confirmed by a markedly reduced pregnancy rate and decreased number of pups per litter, and rapidly progress to sterility by 10 months of age [18].

Histologically, many premature age-related testicular defects occur in the *Lxrαβ* -/- mice and the histological features relate to deranged lipid metabolism with lipid droplets within Sertoli cells of 3.5 month old male *Lxrαβ* -/- mice [18]. By 5.5 months more lipid droplets form in Sertoli cells and also in spermatids and ~20–30% of the seminiferous tubules have cell aggregates with no spermatozoa. By 10 months of age there are mostly empty seminiferous tubules and by 12 months, marked cellular necrosis, larger size lipid droplets and completely disorganised seminiferous tubules are observed [18]. Additionally, basal expression of LXR-regulated genes such as *Srebf1c, Fasn* is decreased by around 40% although no changes are seen in *Srb1*, *Scd1* or *Abca1* expression [18].

Both of the single LXR knockouts are fertile, but a detailed assessment revealed that *Lxrβ* -/- mice have lipid droplets within the Sertoli cells from 2.5 months of age, and *Lxrα* -/- mice have lower levels of intratesticular testosterone and reduced expression of the gene encoding *3β-*Hydroxysteroid dehydrogenase (*3β-HSD)*, important for androgen production [18,66]. Treatment with the LXRα and LXRβ agonist T0901317 restored testosterone levels with increased expression of steroidogenic acute regulatory protein (StAR) enzymes [18,66].

An important recently published study of an additive transgenesis model using *Lxrαβ* -/- mice in which LXRβ was reinstated only in Sertoli cells (driven by the Anti-Mullerian hormone promoter) (*Lxrαβ* -/- *: AMH- Lxrβ*) has provided further interesting information. This ‘rescued’ strain has a reduction in lipid droplets, increased integrity of the BTB and normalisation of testosterone. Thus, information gained from these LXR mouse models has provided new information on the likely roles of LXRs in the testis (summarised Table 1). In human fertility, LXRs are likely to have a similar role to those in the mouse and may be related to premature loss of fertility. Both *LXRα* and *LXRβ* are expressed in human testicular biopsy specimens along with low levels of *SREBP1*c and *IDOL,* both of which are LXR target genes described in men with worse fertility phenotype [74]. However, to date, the specific role of each LXR isoform in the human testis has not yet been elucidated.

## 6. LXRα and LXRβ Control Germ Cell Numbers with Distinct and Overlapping Roles in Both Germ and Somatic Cells of the Testis

Spermatogenesis is tightly regulated process and is hormonally regulated by the hypothalamic-pituitary-gonadal (HPG) axis, with pulsatile secretion of gonadotropins (LH and FSH) leading to androgen synthesis by Leydig cells. Sertoli cell numbers and function dictate germ cell number, with a delicate balance between germ cell proliferation and apoptosis [55]. Successful spermatogenesis requires intact germ:somatic cell and somatic:somatic cell relationships within the testis.

In *Lxrαβ* -/- mice there is increased loss of germ cells by apoptosis and reduced proliferation activity, leading to premature sterility [18,73]. The mechanisms by which this occurs are elusive but interestingly there is compensation between the LXR isoforms. It has been shown that *Lxrα* -/- mice have a significantly higher number of apoptotic cells compared with wild-type mice but this is not the case in the *Lxrβ* -/- mice [18,66]. Interestingly, *Lxrβ* -/- mice exhibit reduced germ cell proliferation which would ordinarily lead to reduced germ cell numbers; however, a compensatory reduction in apoptosis genes ensures little effect on germ cell numbers [18,66]. Ultimately, the marked germ cell loss observed in the *Lxrαβ* -/- mice is likely multifactorial with some direct effect on germ cells. However, with over 40 germ and somatic cell subtypes expressing either LXRα or LXRβ in the adult testis, delineating germ cell expression pattern, deciphering the stages and gauging how LXR may mediate any direct effects on germ cells remains a challenge.

Deregulation of Sertoli cells alone in LXR knockout mice may contribute to germ cell loss, as Sertoli cells are so crucial for all aspects of germ cell development in producing nutrients, growth factors and lipids. Furthermore, their role in maintenance of the BTB, keeping germ cells protected in an immune privileged site, is a crucial one. In *Lxrβ* -/- and *Lxrαβ* -/- mice, Sertoli cells become lipid laden and it can be postulated the large lipid droplets in the adluminal compartment of the testis mechanically disrupt the cytoskeleton, impacting the BTB and culminating in vulnerability of meiotic germ cells and loss of the germ cell pool. Additionally, it is well recognised that Sertoli cell function is important for germ cell lipid homeostasis, but the additive transgenesis model referenced above has shown new insights around this [66]. In this model, where LXRβ is reinstated into Sertoli cells (*Lxrαβ* -/-:*AMH- Lxrβ),* there is an increase in *Abca1, Abcg1* with normalisation of intracellular cholesterol levels in the testis. However, a persistence of lipid inclusions in spermatids suggests that, in these cells, regulation of lipids may be independent of the LXRβ activity in Sertoli cells [66].

Other somatic cells, such as Leydig, cells are important for germ cell function and numbers, and the low testosterone described in both the *Lxrα* -/- and *Lxrαβ* -/- mice will affect germ cell development since adequate testosterone and functioning androgen receptor are required for spermatogenesis maintenance [75,76]. Furthermore LXRs regulate inhibin and FSH levels, and low FSH levels are observed in *Lxrαβ* -/- mice which may influence spermatogonial number, entry into meiosis, and have indirect effect on androgen production on Leydig cells [77].

## 7. LXRα Regulates Testosterone Production but Requires Cooperation from LXRβ-Expressing Sertoli Cells

**Figure 2:**
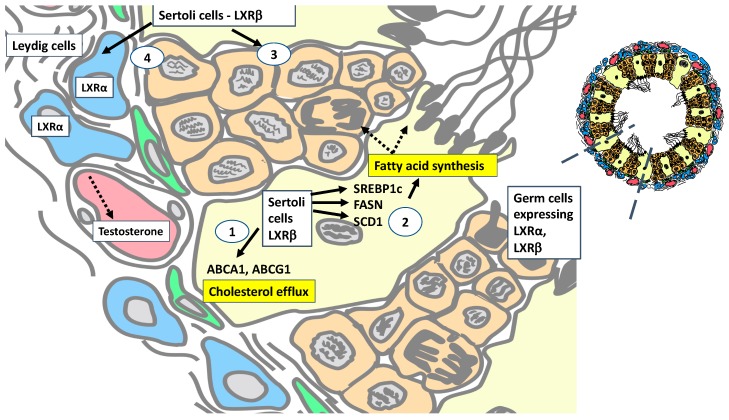
Schematic representation of seminiferous tubule and interstitium illustrating main roles of LXRs in the testis. LXRα is expressed in Leydig cells and LXRβ is expressed in Sertoli cells. Male germ cells express both isoforms. LXRβ regulates expression of genes important for lipid homeostasis processes such as (1) cholesterol efflux notably ABC transporters ABCA1, ABCG1 which reduce cellular cholesterol levels (2) fatty acid synthesis genes SREBP1c, SCD1, FASN and fatty acids. which are used by Sertoli cells but also maturing germ cells as fuel (3) LXRβ is important for maintenance of the blood testis barrier and (4) LXRβ regulates the endocrine function of Leydig cells.

Androgens are mainly produced by the Leydig cells of the testis and it is recognised that the LXR agonist T0901317 induces a 13-fold increase in intratesticular testosterone [18]. Studies using *Lxrα* -/- mice revealed diminished steroidogenic activity of Leydig cells and low testosterone levels. Central endocrine function was also affected, with reduced mRNA expression of the *β* chain of the luteinizing hormone (*Lh)* receptor in the pituitary and decreased follicle stimulating hormone (FSH) in both *Lxrα* -/- and *Lxrαβ* -/- mice [18]. In the *Lxrαβ* -/-:*AMH- Lxrβ* transgenic mouse model, normalisation of intratesticular testosterone and genes encoding steroidogenic enzymes *Star* and *3β-hsd1* and partial restoration of FSH occur [66]. Thus, restoration of LXRβ in Sertoli cells normalised steroidogenesis in Leydig cells independent of the hypothalamus or pituitary gland (Figure 2). This suggested a paracrine interaction between Sertoli and Leydig cells driven by LXRβ that restores androgen levels, highlighting the cooperation between Sertoli and Leydig cell in terms of endocrine function [66].

However, there are persistently low LH levels in these mice, suggesting a remaining central defect in LH secretion not rescued by reintroduction of Lxrβ into Sertoli cells. The hypothalamus also expresses both *Lxrα* and *Lxrβ*, and pharmacological LXR activation with GW3965 leads to GnRH and gonadotrophin responses [78,79].

Together, this all suggests that LXRs are important for androgen synthesis and the low testosterone levels observed in *Lxrαβ* -/- mice [18] may be due to effects at multiple levels of the HPG axis, but normalisation of androgens can occur if LXRβ is reintroduced in Sertoli cells [66].

## 8. The Role of LXRs in Post-Testicular Development Events in the Epididymis

Although LXRs have an important role in maintaining epididymal function, the ‘infertility’ phenotype of the *Lxrαβ* -/- mice is a composite of sequelae from testicular and epididymal dysfunction. While LXRβ is expressed throughout the epididymis, LXRα is expressed in only some regions of the epididymis [80]. Unlike in the testis, there are no studies exploring LXR signalling in human epididymis; however, rodent studies suggest that defective LXR signalling leads to an imbalance in cholesterol metabolism, alterations in proliferation/apoptosis, and production of proinflammatory mediators in the epididymis [81,82,83].

Loss of LXRs affects normal functioning of the epididymal epithelial cells required for crucial lipid modifications to spermatozoa membranes during epididymal transit. The epithelial epididymal cells in *Lxrαβ* -/- mice are shrunken, with cholesteryl ester accumulation, an enlarged lumen of epididymal tubules (particularly the first 2 epididymal segments) with the presence of an amorphous substance and features similar to atherosclerosis [81].

The epididymis acts as a quality control organ, removing abnormal sperm as they transit, but when epididymal epithelial cells are dysfunctional, sperm is also affected. There is increased midpiece fragility observed in *Lxrαβ* -/- mice and the presence of isolated sperm heads and flagella [81]. Recent studies show that the epididymis from *Lxrβ* -/- mice had significantly lower levels of *Srebf1, Fasn, Abca1* (likely resulting in the aforementioned cholesteryl ester deposition)*, Abcg1 and Idol* expression [81,82,83]. In addition, lower sperm counts and more broken sperm tails were seen in these mice [81,82,83].

Recently, studies challenging 4-month-old *Lxrαβ* -/- mice with a high cholesterol diet (HCD) revealed accelerated changes in cholesterol deposition within the epididymis [84] with lipid deposition in the smooth muscle cells (SMCs) surrounding the epididymal tubules [70,83]. Epididymal SMCs in the *Lxrαβ* -/- mice transdifferentiate into macrophage-like foam cells by 9 months of age or at 4 months if fed a HCD [70,83], similar to the situation seen in atherosclerosis [85]. Characterisation of sperm lipids from the *Lxrαβ* -/- HCD challenged mice revealed problems with sperm, with higher cholesterol:phospholipid ratios, and subsequent negative effects on the capacitation and fertilisation potential [84].

## 9. LXR Loss Is Also Associated with a Pro-Inflammatory State Potentially Affecting Fertility

Chronic testicular inflammation may result from a variety of causes which include infection, toxic insults, metabolic diseases and obesity, all of which are associated with enhanced production of proinflammatory cytokines and reactive oxygen species (ROS). This causes oxidative stress affecting male fertility [86,87,88]; ROS production is associated with increased germ cell apoptosis, DNA fragmentation and altered fluidity of the sperm membrane [89]. Measuring oxidative stress (OS) when assessing male infertility is one of the more recent clinical recommendations [90]. Oxidative stress is also associated with high levels of interleukin-6 (IL-6), a proinflammatory cytokine associated with unfavourable semen parameters [91].

LXRs exert anti-inflammatory effects, both direct and indirect, through transactivation or trans-repression mechanisms [5,6]. LXRs negatively regulate the expression of NF-kB dependent proinflammatory cytokines such as IL-6 [92], while SUMOylated LXR can tether corepressors to promotor sites of genes important in inflammatory responses, thus exerting anti-inflammatory effects [5,93]. LXRs also modulate Toll-like receptor 4 (TLR4) signalling, and LXR activation causes ABCA1-mediated changes in membrane lipids, disrupting the MydD88 and TRAF6 recruitment required for NF-kB signalling, therefore inhibiting production of proinflammatory cytokines [5,93].

Another potential role for LXRs in immunomodulation in the testis may occur in testicular macrophages, which are rarely studied and help to maintain testicular immune privilege. LXR-dependent processes in macrophages may mediate some of the anti-inflammatory effects in the testis, and IL-5-mediated reprogramming of macrophages is one of the immunomodulatory roles of LXRs [94,95]. Additionally, LXRs may reduce NF-kB signalling and reduce levels of the proinflammatory cytokines IL-6 and TNFα [96]. Support for this comes from studies of the epididymis from *Lxrα* -/- and *Lxrβ* -/- mice. These mice have increased epididymal inflammation and altered epithelial cell function; *Lxrα* -/- mice have increased expression of *Il-1β* and *Tnfα* whilst *Lxrβ* -/- mice have increased expression of *Il-6* and *Tnfα* genes, which are associated with inflammation.

## 10. Conclusions

LXRs typically upregulate a suite of genes crucial for cholesterol balance and phospholipid remodelling with anti-inflammatory effects. In the testis and epididymis, strict lipid homeostasis is required for normal fertility: conditions associated with lipid disorders such as obesity, hypercholesterolaemia are increasingly recognised as being associated with male subfertility. Extensive rodent studies to date reveal that LXRs are crucial for male fertility, and *Lxrαβ* -/- mice become prematurely infertile due to combined problems in the testis and the epididymis. More data are required to explore the roles of LXRs in the human testis, in particular, interrogation of differences in fertility phenotypes.

## Figures and Tables

**Table 1:** Summary of defects reported in LXR knockout mice [18,73] and effects of rescuing Lxrβ in Sertoli cells [66].

Genotype	Fertile	Abnormalities/Comments	
*Lxrα* -/-	yes	Low testosterone levels Normal germ cell numbers	
*Lxrβ* -/-	yes	Sertoli cells: cholesterol deposition Normal germ cell numbers	
*Lxrαβ* -/-	5 months of age then infertile	3.5 months	Lipid droplets in Sertoli cells Larger Leydig cells
		5.5 months	20–30% abnormal seminiferous tubules Cell aggregates not spermatozoa
		10 months	Empty tubules Lipid droplets ++ in Sertoli cells
		12 months	Completely disorganized testis, cholesterol deposition Numerous vacuoles in interstitial and seminiferous tubules.
*Lxrαβ* -/- with rescue *Lxrβ* :AMH	Unknown	Normalised intratesticular testosterone and follicle stimulating hormone (FSH) levels with Lxrβ but abnormally low luteinizing hormone (LH) Restored lipid homeostasis in Sertoli cells but persistence of lipid inclusions in spermatids Accumulation of neutral lipids ion peritubular myoid cells	
